# A nationwide analysis of 350 million patient encounters reveals a high volume of mental-health conditions in primary care

**DOI:** 10.1038/s44220-024-00310-5

**Published:** 2024-09-19

**Authors:** Avshalom Caspi, Renate M. Houts, Terrie E. Moffitt, Leah S. Richmond-Rakerd, Matthew R. Hanna, Hans Fredrik Sunde, Fartein Ask Torvik

**Affiliations:** 1 Department of Psychology and Neuroscience, Duke University, Durham, NC USA; 2 Department of Psychiatry and Behavioral Sciences, Duke University School of Medicine, Durham, NC USA; 3 Institute of Psychiatry, Psychology, and Neuroscience, King’s College London, London, England; 4 Promenta Research Center, University of Oslo, Oslo, Norway; 5 Department of Psychology, University of Michigan, Ann Arbor, MI USA; 6 Centre for Fertility and Health, Norwegian Institute of Public Health, Oslo, Norway

**Keywords:** Epidemiology, Health services, Psychiatric disorders

## Abstract

How many primary-care encounters are devoted to mental-health conditions compared with physical-health conditions? Here we analyzed Norway’s nationwide administrative primary-care records, extracting all doctor–patient encounters occurring during 14 years (2006–2019) for the population aged 0–100 years. Encounters were recorded according to the International Classification of Primary Care. We compared the volume of mental-health encounters against volumes for conditions in multiple different body systems. A total of 4,875,722 patients generated 354,516,291 encounters. One in 9 encounters (11.7%) involved a mental-health condition. Only musculoskeletal conditions accounted for a greater share of primary-care physicians’ attention. The volume of mental-health encounters in primary care equaled encounters for infections, cardiovascular and respiratory conditions and exceeded encounters for pain, injuries, metabolic, digestive, skin, urological, reproductive and sensory conditions. Primary-care physicians frequently treat complex mental-health conditions in patients of every age. These physicians may have a more important role in preventing the escalation of mental-health problems than heretofore appreciated.

## Abstract (web summary)

The authors present findings of more than 350 million primary-care visits over 14 years in the Norwegian healthcare system, indicating that 1 in 9 encounters involved a mental-health condition and peaking at age 40 years (18.7%).

## Main

Primary-care physicians (PCPs) are important providers in many healthcare systems. PCPs treat all common health conditions, both acute and chronic; they provide preventive care and health education to patients; and they refer some patients to specialist treatment. Although many PCPs feel ill-equipped to deliver care that is the province of psychiatric services^1^, they are often the first point of contact for navigating mental-health care. Current efforts to strengthen primary care aim to address mental health and make whole-person care a reality^2,3^. An accurate account of what PCPs are managing and treating is needed to inform medical education and healthcare delivery. It is important to know the volume and types of mental-health conditions for which patients present to their PCPs, to ensure that PCPs are well trained to deliver evidence-based treatments to patients and so that healthcare systems are positioned to integrate mental-health services into primary-care settings.

How much of PCPs’ work is devoted to addressing mental-health conditions? The available information can be confusing. For example, a report from the American Psychological Association claims that ‘as many as 70% of primary-care visits are driven by patients’ psychological problems’^4^ and a survey carried out by the UK charity Mind reports that 40% of PCPs’ appointments involve mental health^5^. Some of the most thorough data about the volume of mental-health concerns addressed in primary-care visits come from the National Ambulatory Medical Care Surveys, which provide information about care provision in the United States obtained via physician surveys^6,7^. These data indicate that the percent of primary-care visits with a mental-health concern as a primary diagnosis increased from 3.4% of visits in 2006–2007 to 6.3% by 2016–2018. However, these data have some limitations. The survey response rate is variable (<50% in 2018)^8^; the care-provision observation window is a randomly selected 1-week reporting period; and the information is restricted to patients over age 18 years. With increasing concerns about population mental health^9^, the goal of our article is to provide information that can inform discussion of useful ways to address mental-health needs.

**Fig. 1:**
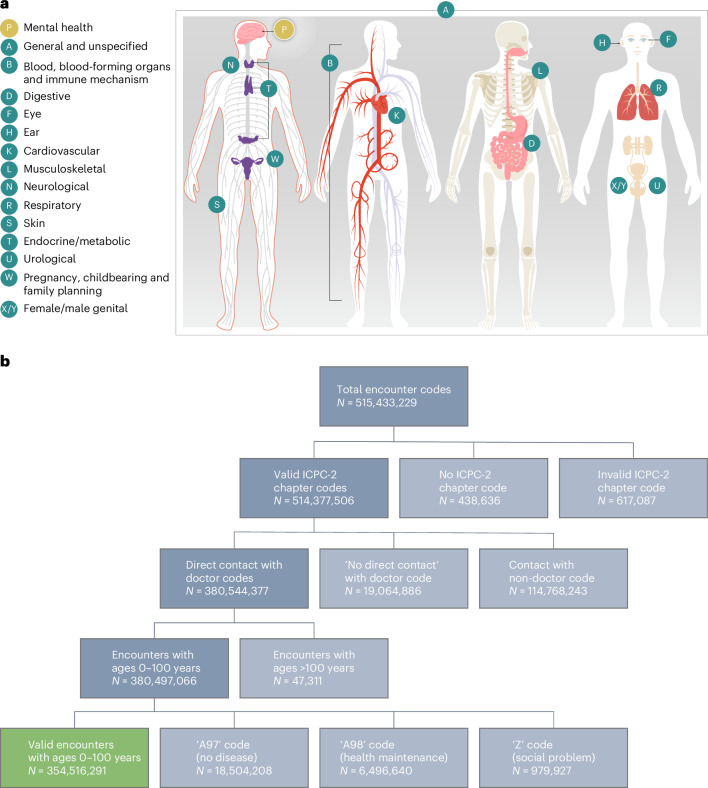
An analysis of primary-care encounters. **a**, The International Classification of Primary Care (ICPC-2), which focuses on conditions that are encountered in primary care and which are coded into chapters representing body systems. **b**, The flow chart of medical conditions studied in this nationwide analysis of health encounters in general practices.

To provide information about the volume of mental-health conditions in primary care, we turned to a unique data source: all primary-care records in the health system of an entire nation where cost barriers do not generate bias in the subset of unwell individuals who seek care. These are administrative data from Norway, where all residents are assigned a PCP. To our knowledge, this is the only nationwide source of data about mental health in primary-care settings with complete coverage of the entire population of a nation. We extracted all codes associated with each patient encounter over a 14-year period from 2006 to 2019 for the patient population aged 0–100 years. These data allowed us to estimate (a) how much of PCPs’ work is devoted to addressing mental health, (b) the volume of the different types of mental-health conditions that PCPs are called on to address, (c) the volume of mental-health conditions that PCPs address at different stages of their patients’ lifespan, and (d) to compare estimates of PCPs’ mental-health encounters against estimates of encounters for other medical conditions. Specifically, we compared the volume of PCPs’ mental-health encounters against estimates of PCPs’ encounters for medical conditions in 15 different body systems (for example, skin, digestive, eye and cardiovascular), as coded according to the International Classification of Primary Care (ICPC)^10^, as well as against encounters for body-wide infections, pain and injuries (Fig. 1). The result is a unique portrait of the workload of PCPs, with implications for how to strengthen primary care.

## Results

### Proportion of patients who present to PCPs for mental-health conditions

**Fig. 2:**
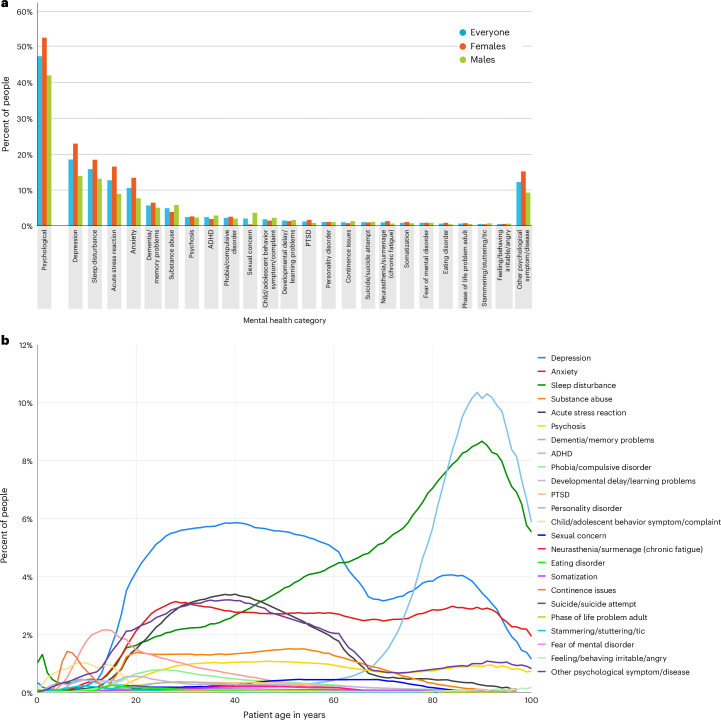
Patient-level analysis: proportion of patients who present to PCPs with mental-health conditions. **a**, The proportion of patients presenting with different mental-health conditions (Supplementary Table 4). **b**, The distribution of mental-health conditions over the lifespan, as a function of age.

Nearly half (47%, *N* = 2,309,787) of the 4,875,722 people registered with a PCP between 2006 and 2019 presented to primary care for a mental-health condition, as recorded by their PCPs. These patients experienced the full range of psychological difficulties, from depression to irritability/anger (Fig. 2a and Supplementary Table 4). Children were most likely to attend primary care for sleep disturbances, continence issues and ADHD; young and middle-aged adults for depression; and older adults for memory difficulties and, once again, sleep disturbances (Fig. 2b).

### Proportion of PCP encounters devoted to mental health

**Fig. 3:**
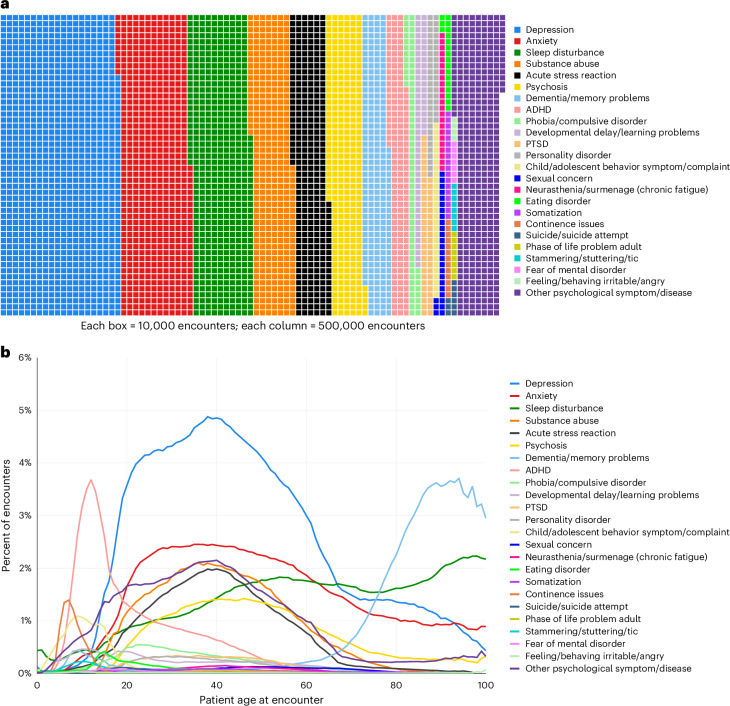
Encounter-level analysis: proportion of primary-care encounters devoted to mental health. **a**, The proportion of 41,616,704 mental-health encounters devoted to each of 24 different mental-health conditions (Supplementary Table 5). **b**, Mental-health encounters generated by patients at every point over the lifespan.

Patient-level analyses reveal the proportion of patients who present in general practices for mental-health conditions, but patient-level analyses are not informative about the volume of patient encounters that PCPs devote to mental-health conditions. To address this question, we analyzed primary-care encounters as the unit of analysis. The 4,875,722 people registered with a PCP generated 354,516,291 encounters with their PCPs between 2006 and 2019. Of all primary-care encounters, 11.7% involved a mental-health condition (*N* = 41,616,704). As shown in Fig. 3a (Supplementary Table 5), of the 41,616,704 mental-health encounters, over one-third involved depression (23.8%) or anxiety (14.1%), followed by sleep disturbances (12.1%), substance abuse (8.3%), acute stress reactions (7.1%), psychosis (6.9%), dementia/memory problems (5.4%), ADHD (3.8%), phobias/compulsive disorders (1.7%), developmental delay/learning problems (1.5%), PTSD (1.3%) and personality disorder (1.1%). Under 1% each involved child and adolescent behavior problems/complaints, sexual concern, neurasthenia/surmenage (chronic fatigue), eating disorder, somatization, continence issues, suicide/suicide attempt, phase of life problem in an adult, stammering/stuttering/tic, fear of mental disorder and feeling/behaving irritable/angry. One out of every 11 mental-health encounters (9.1%) was unspecified and coded by PCPs as psychological symptoms/complaints or ‘other’ disorders. Mental-health encounters were generated by patients at every stage of the lifespan (Fig. 3b).

### Mental-health encounters versus encounters for other conditions

**Fig. 4:**
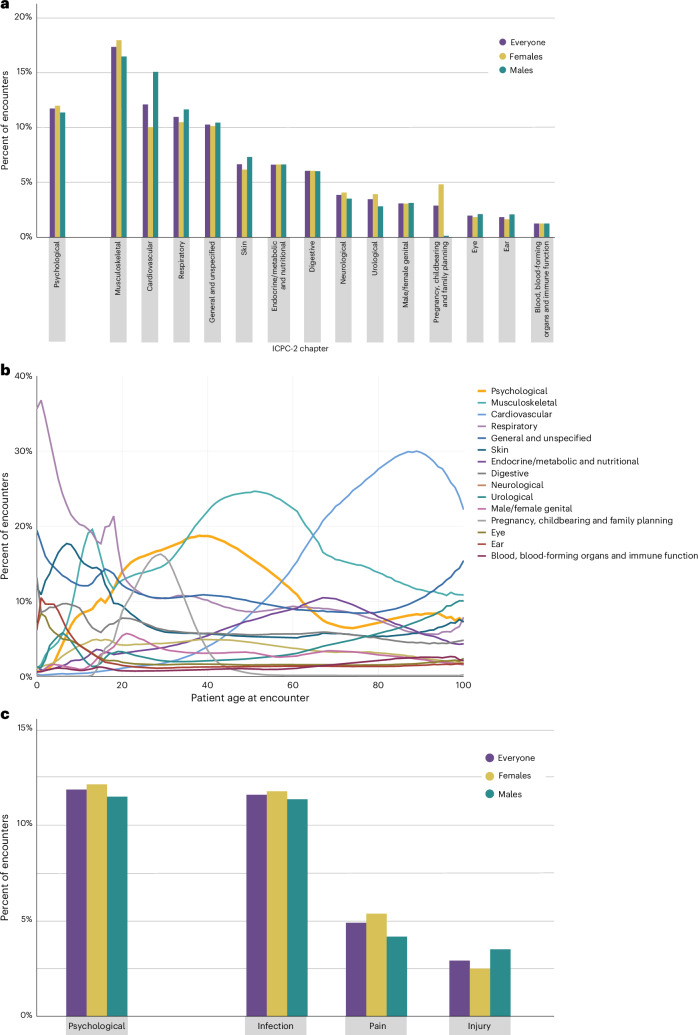
How does the number of primary-care encounters for mental-health conditions compare to the number of encounters for other conditions that PCPs see in their practice?. **a**, Comparison of the volume of PCPs’ mental-health encounters to encounters for medical conditions in 15 different body systems (Supplementary Table 6). **b**, Comparison of the volume of PCPs’ mental-health encounters to encounters for medical conditions in 15 different body systems, over the lifespan as a function of patient age. **c**, Comparison of the volume of PCPs’ mental-health encounters to encounters for infections, pain and injuries, throughout the body (Supplementary Table 7).

Table 1 shows the distribution of encounters for conditions in each body system, across the population of patients. Figure 4a (Supplementary Table 6) compares the volume of PCPs’ mental-health encounters to PCPs’ encounters for medical conditions in 15 different body systems (for example, musculoskeletal, skin, digestive, eye and cardiovascular). Of all encounters in primary-care settings, 11.7% were for mental-health conditions. The only body system that accounted for a greater share of PCPs’ attention was the musculoskeletal system (17.4% of all encounters). Mental health (11.7%) accounted for a similar share of encounters as the cardiovascular system (12.1%) and the respiratory system (11.0%), as well as general/unspecified conditions (10.3%). PCPs had two to nine times more mental-health encounters than encounters for conditions in every other body system (that is, skin, endocrine/metabolic, digestive, neurological, urological, male/female genital, pregnancy/childbearing/family planning, eye, ear and blood/blood-forming organs/immune function). We further examined 31 common physical disorders grouped within nine broad categories (circulatory, endocrine, pulmonary, gastrointestinal, urogenital, musculoskeletal, hematologic, and neurologic conditions and cancer). PCPs had more mental-health encounters than they did encounters for common disorders ranging from circulatory disorders to cancer (Supplementary Fig. 1).

**Table 1:** A nationwide analysis of patient encounters in general practices in Norway from January 2006 through December 2019

		A. Patients		B. Encounters						C. Percent of total encounters for a chapter code accounted for by *X*% of patients with the chapter code			
						Encounters per person							
Chapter code and corresponding body system		*N*	%	*N*	%	Mean	s.d.	50%	99%	10%	20%	30%	40%
A	General and unspecified	4,057,779	83.2%	36,372,091	10.3%	8.96	17.64	5	66	44.5%	61.3%	72.5%	80.7%
B	Blood, blood-forming organs and immune function	772,099	15.8%	4,438,999	1.3%	5.75	12.29	2	56	53.0%	68.8%	78.1%	84.3%
D	Digestive	3,087,194	63.3%	21,400,200	6.0%	6.93	12.12	3	52	45.1%	62.2%	73.4%	81.3%
F	Eye	2,360,784	48.4%	6,942,772	2.0%	2.94	3.44	2	16	34.9%	51.5%	63.3%	72.8%
H	Ear	1,957,172	40.1%	6,431,506	1.8%	3.29	4.16	2	19	36.7%	53.8%	65.5%	74.6%
K	Cardiovascular	2,069,201	42.4%	42,926,196	12.1%	20.75	35.60	8	184	50.1%	68.9%	80.7%	88.4%
L	Musculoskeletal	3,846,867	78.9%	61,516,933	17.4%	15.99	25.47	7	118	46.2%	65.2%	76.9%	84.8%
N	Neurological	1,994,268	40.9%	13,633,579	3.8%	6.84	15.52	3	69	54.4%	69.9%	79.0%	85.4%
P	Psychological	2,309,787	47.4%	41,616,704	11.7%	18.02	39.72	6	176	54.9%	72.4%	82.6%	89.0%
R	Respiratory	4,046,751	83.0%	38,917,254	11.0%	9.62	13.46	6	61	39.7%	57.3%	69.5%	78.6%
S	Skin	3,836,601	78.7%	23,559,554	6.6%	6.14	8.43	4	36	37.3%	54.4%	66.7%	75.9%
T	Endocrine/metabolic and nutritional	1,924,621	39.5%	23,483,464	6.6%	12.20	20.03	4	95	49.5%	69.5%	80.7%	87.6%
U	Urological	1,888,144	38.7%	12,211,775	3.4%	6.47	11.15	3	52	47.0%	64.3%	75.0%	82.4%
W	Pregnancy, childbearing and family planning	875,252	18.0%	10,179,018	2.9%	11.63	12.88	6	55	35.5%	56.8%	71.8%	82.0%
X/Y	Male/female genital	2,029,104	41.6%	10,886,246	3.1%	5.37	9.13	3	43	45.2%	61.7%	72.6%	80.5%
	Any diagnosis	4,831,723	99.1%	354,516,291	100.0%	73.37	84.32	47	388	35.9%	54.4%	67.5%	77.2%

Panel A shows the number of patients who presented to PCPs with different medical conditions. Panel B shows the volume of health encounters that PCPs encountered, along with the distribution of encounters generated by patients. Panel C shows the concentration of health encounters among patients.

Notes: The table shows the number of patients receiving primary care, out of 4,875,722 individuals born in Norway from February 1905 through December 2017 who were full-time residents in Norway from January 2006 until December 2019. Medical conditions are coded according to chapters in the International Classification of Primary Care, 2nd edition (ICPC-2). ICPC includes 17 chapters. We present data for 16 chapters representing body systems. We excluded the 17th chapter Z, which refers to Social Problems (Fig. 1b).

Figure 4b compares the volume of PCPs’ mental-health encounters to encounters for conditions in 15 different body systems, as a function of patient age. Encounters for respiratory-system conditions were most common when treating children, and encounters for cardiovascular-system conditions were most common when treating older adults. When treating young-adult to middle-aged patients between the ages of 20 and 60 years, encounters for mental-health conditions vied with the musculoskeletal system for the most attention from PCPs. For example, with 40-year-old patients, nearly 2 in 10 (18.7%) encounters involved a mental-health condition and another 2 in 10 (22.0%) involved a condition in the musculoskeletal system.

We also compared the volume of mental-health encounters to encounters for infections, pain and injuries, all occurring anywhere in the body. Figure 4c (Supplementary Table 7) shows that PCPs had an equal number of encounters for mental-health conditions (11.7% of all encounters) as they did for infections (11.5%). PCPs had 3–4 times more encounters for mental-health conditions than they did for symptoms/complaints of pain (4.8%) or injuries (3.8%).

## Discussion

Alarms about poor mental health have been sounded by governments, non-governmental organizations and on social-media platforms. There is now a month, a week and a day devoted to raising mental-health awareness^11–13^. And yet, there continues to be concern about the availability of and access to resources for confronting the challenge of population mental health^14,15^. This concern is underscored by the present analysis, which reveals the magnitude of PCP work devoted to mental-health conditions in a nation’s primary-care settings.

We analyzed over 350 million nationwide encounters in general practices gathered over the course of 14 years in Norway. Three findings stand out. First, the volume of mental-health encounters in primary-care practices is large. One out of every 8 or 9 encounters that a PCP has is for a mental-health concern. Only musculoskeletal conditions account for a greater share of PCPs’ attention. The volume of PCPs’ mental-health encounters is equal to their encounters for the cardiovascular and respiratory systems and exceeds encounters for the metabolic, digestive, skin and sensory systems. It is also striking that PCPs have as many encounters for mental-health conditions as they do for infections, and many more mental-health encounters than encounters for pain and injuries. Second, mental-health encounters in primary care are not just limited to a few of the most common mental disorders. About one-third of mental-health encounters involve depression or anxiety, but the rest include diverse and complex mental-health complaints, symptoms and disorders. Third, mental-health encounters are generated by patients at every stage of the lifespan. Many of these encounters are for developmentally specific conditions, such as children’s learning problems or older adults’ memory problems. However, others are for conditions prevalent in all age groups (for example, phobias and depression) whose treatment delivery must be tailored to patients’ age-related cognitive capacities and life circumstances.

On the one hand, the volume of mental-health encounters may be overestimated here because sleep disturbances and dementia are included as codes in chapter P-Psychological of ICPC-2. But their classification as psychological conditions has merit, as effective treatments for sleep disturbances include cognitive and behavioral treatments^16^, and dementia is a neurocognitive disorder. On the other hand, the volume of mental-health encounters may be underestimated because pain codes and codes that identify patients’ fears of medical conditions—both of which have psychological elements—are not listed as mental-health encounters, but are instead included alongside their corresponding body system in the ICPC-2 coding system. In addition, our data were limited to a visit’s primary diagnosis, as submitted by PCPs for reimbursement. Data from physician-surveys that collect information about additional diagnoses or reasons associated with a visit beyond the primary diagnosis suggest that the volume of mental-health conditions associated with primary-care visits may be two to three times greater than the volume estimated by the primary diagnosis alone^7^. More generally, the classification of mental-health conditions is challenging in primary care^17^. Inter-doctor and inter-practice variation influence data precision, and limited time with patients may lead to misclassification. It is possible that there is misclassification of some mental disorders (for example, misclassification between depression and anxiety), and PCPs may also use ICPC-2 P-chapter codes as ‘placeholder’ conditions when they are uncertain of which mental-health condition to assign. This may affect estimates of the volume of specific mental-health conditions, but not the overall volume. ICPC-2, like other mental-health diagnostic systems, is imperfect. However, it has face validity to the patient seeking care and to the physicians who are reporting these codes. Moreover, comparisons of mental-health diagnoses made by Norwegian PCPs and by clinical psychologists administering psychiatric interviews reveal the same etiological risk factors^18^.

This analysis has several strengths. First, we drew on a unique data resource: all primary-care records in an entire nation. Because all residents in Norway are assigned a PCP, the dataset was not biased by selective inclusion of primary-care practices and was sufficiently large to identify even rare conditions. Second, we focused on PCPs because they are gatekeepers in many healthcare systems and often help patients navigate mental-health care (for example, 97.3% of individuals in Norway with psychiatric codes in specialist healthcare also had an ICPC-2 P-code in primary care). Third, because a diagnosis of a mental disorder is not always appropriate or cannot be made accurately in primary-care settings, we deliberately included symptoms and complaints that served as the impetus for referral or help-seeking, and that required PCPs’ attention. Importantly, when we restricted our analysis to diagnosed mental disorders, the volume of mental-health encounters in primary care still exceeded encounters for eight other broad categories of medical disorders, excepting only circulatory disorders.

This analysis has limitations. First, generalizability outside Norway may be limited owing to different healthcare and socioeconomic structures. Second, the ICPC-2 psychological codes capture the reason the patient is seeking medical care. They should not be interpreted as prevalence rates of mental-health conditions in the population, but as population-level estimates of service contacts for mental health. Third, we do not have treatment information about each encounter, as is available in physician-survey data^7^, and we cannot evaluate how PCPs cared for mental-health conditions. Fourth, we analyzed primary-care encounters over a 14-year period ending in 2019. During this period, 11.7% of encounters involved a mental-health condition, ranging from 11.0% in 2006 to 12.8% in 2019. This volume cannot be attributed to the COVID-19 pandemic, which triggered an increase in depression, anxiety and substance-use problems^19^. The volume detected here may underestimate the extent of mental-health care provided by PCPs after 2019.

These findings have implications for medical training, treatment and public understanding of mental health. First, PCPs need to be trained to be mental-health generalists. PCPs are not only encountering depression and anxiety. Mental-health encounters in primary care are diverse, range from mild to moderate to severe, and span pediatric to geriatric conditions. Learning to manage this panoply requires dedicated mental-health curricula alongside more trained teachers^20,21^. One impetus for increased mental-health education is that there are calls for PCPs to provide more advanced mental-health care, given the shortage of specialist psychiatrists and psychologists. Another impetus is that PCPs who handle mental-health encounters for which they feel ill-prepared may be especially susceptible to burnout, an occupational syndrome driven by mismatches between workload, effort and reward, with costly effects^22–24^. For example, turnover among American PCPs is estimated to result in nearly one billion dollars in excess healthcare expenditures annually, more than one-fourth of which has been attributed directly to physician burnout^22^. Increasing knowledge about mental-health conditions and feelings of preparedness to treat them may make the workload more manageable.

Second, our findings support calls for integrating mental-health services into primary-care settings^25^. An important component of this integration is facilitating linkage to specialty mental-health care, such as via ‘warm handoffs’^26^. Mental-health training for physician assistants and increased training of clinical psychologists in primary care may also be needed to create a primary-care workforce that can deliver both mental-health and physical-disease care^27,28^. Our volumetric analysis does not speak to policy choices, but underscores that PCPs shoulder a large demand for mental-health care, which may be difficult to deliver especially when this calls for evidence-based nonpharmacological treatments.

Third, our findings may help to further normalize help-seeking^29,30^. Even in the relatively brief 14-year period studied here, half of the population presented to their PCPs with a mental-health problem. Over the life course, a majority of individuals will consult their PCP for a mental-health problem. It is critical for the mental health of the population that PCPs are well equipped to meet this demand. As the first line of treatment, PCPs often encounter less severe conditions and could have the opportunity to prevent these problems from escalating.

Mental-health conditions are believed to be on the rise around the world, but effective treatment coverage remains low^31^. Current efforts to strengthen primary care aim to address mental health and make whole-person care a reality. This study adds information about the magnitude of mental-health care in primary care. The large volume of primary-care encounters devoted to diverse mental-health conditions underscores the need for physician training in mental health, for integrated mental-health services and for workforce capacity planning. PCPs may have a more important role in preventing the escalation of mental-health problems than heretofore appreciated.

## Methods

### Study population

This population-based study included 4,875,722 individuals (2,433,978 males; 2,441,744 females) born in Norway between February 1905 and December 2017 who were full-time residents in Norway from January 2006 until December 2019 or until they died, as identified in the Norwegian Population Register.

Ethics approval was approved from the Regional Committee for Research Ethics South East Norway (REK South East) and the Arts and Sciences Institutional Review Board at Duke University (2022-0260).

### Ascertainment of primary-care encounters

All residents of Norway are assigned a PCP. Access to specialist healthcare typically requires a referral from the PCP. Service is free for juveniles and highly subsidized for adults. To receive reimbursements, PCPs bill the Norwegian Health Economics Administration, sending at least one primary diagnosis or reason for the visit. Thus, it is unlikely that visits to PCPs go unreported. On average, each visit yielded 1.18 (s.d. = 0.47) codes. The majority of visits (84.5%) yielded one code; 13.9% two codes; and 1.6% 3 or more codes.

Information is coded according to the International Classification of Primary Care (ICPC-2)^10^ and registered in a national database. ICPC-2 is divided into 17 chapters representing health conditions in different body systems (Fig. 1a and Supplementary Table 1).

We had access to data covering information from January 2006 to December 2019. We extracted all PCP visits for patients between ages 0 and 100 years. We included codes associated with contacts between a PCP and a patient, including office visits, telephone contacts and home visits (Fig. 1b).

#### Mental-health conditions

Using codes from ICPC-2 chapter P-Psychological, we grouped mental-health conditions into 24 categories (Supplementary Table 2).

#### Medical conditions in other body systems

We compared mental-health conditions against conditions comprising each of the 15 ICPC-2 chapters representing other body systems: B-Blood/Blood-Forming Organs/Immune Mechanism; D-Digestive; F-Eye; H-Ear; K-Cardiovascular; L-Musculoskeletal; N-Neurological; R-Respiratory; S-Skin; T-Endocrine/Metabolic/Nutritional; U-Urological; W-Pregnancy/Childbearing/Family Planning; X/Y-Female/Male Genital; and A-General and Unspecified (we removed codes A97 (no disease, <5% of all codes) and A98 (health maintenance/prevention, <2% of all codes)). We excluded codes that appear in the 17th ICPC-2 chapter Z-Social Problems (<0.5% of all codes) (Fig. 1b).

#### Infections, pain and injuries

We also compared mental-health conditions against infections, symptoms/complaints of pain and injuries, as PCPs often manage these concerns. Because infections, pain and injuries are distributed throughout different chapters in the ICPC-2 according to the body system they involve, we pooled the relevant codes across the different chapters (Supplementary Table 3).

### Statistical analysis

We report analyses based on two units of measurement: the patient and the encounter.

To estimate the prevalence of patients reporting mental-health conditions when attending primary-care practices, we divided the number of patients with mental-health conditions by the total population of patients visiting PCPs over the 14-year observation period, as a function of patient sex and age.

To estimate the proportional volume of PCPs’ encounters devoted to mental-health conditions, we divided the number of encounters for mental-health conditions by the number of all total encounters recorded by PCPs over the 14-year observation period, as a function of patient sex and age. We then compared the proportion of mental-health encounters to the proportion of medical conditions in 15 body systems and to infections, pain and injuries.

The goal of this analysis is to ascertain the volume of encounters in primary-care settings that are devoted to mental health. We report descriptive statistics and provide data visualizations to summarize primary-care encounters in the population. Analyses were performed in SAS v9.4 TS Level 1M4; graphs were created in R using plotly (v4.9.2.2)^32^. Given the very large number of encounters in the population (>350,000,000), statistical-significance tests are not informative and inferential statistics are not reported.

### Reporting summary

Further information on research design is available in the Nature Portfolio Reporting Summary linked to this article.

## Supplementary information

Supplementary Information Supplementary Tables 1–10, Fig. 1 and references.

Reporting Summary

## Data Availability

The data for this study are primary-care records for entire cohorts of the Norwegian population. Researchers can access the data by application to the Regional Committees for Medical and Health Research Ethics and the data owners (Statistics Norway and the Norwegian Directorate of Health). The authors cannot share these data with other researchers. However, other researchers can contact the authors if they have questions concerning the data.

## Code availability

Code is available at https://moffittcaspi.trinity.duke.edu/research-topics/statistical-code.
