# Supplementary material for: A nationwide analysis of 350 million patient encounters reveals a high volume of mental-health conditions in primary care
Source: Nat Ment Health. 2024 Sep 19;2(10):1208–16. doi: 10.1038/s44220-024-00310-5 (PMC11479939; doi:10.1038/s44220-024-00310-5)
Supplement: Supplementary file 1 — Supplementary Tables 1–10, Fig. 1 and references. [file 44220_2024_310_MOESM1_ESM.pdf]

nature mental health

Supplementary information

https://doi.org/10.1038/s44220-024-00310-5

A nationwide analysis of 350 million patient encounters reveals a high volume of mental-health conditions in primary care

In the format provided by the authors and unedited

Table of Contents

Table S1. International Classification of Primary Care (ICPC), 2nd edition 2
Table S2. ICPC-2 codes for mental-health conditions 4
Table S3. ICPC-2 codes for infections, pain, and injuries 6
Table S4. Proportion of patients presenting with different mental-health conditions. 8
Table S5. Proportion of mental-health encounters devoted to different mental-health conditions. 9
Table S6. Comparison of the volume of PCP's mental-health encounters to encounters for medical conditions in 15 different body systems. 10
Table S7. Comparison of the volume of PCP's mental-health encounters to encounters for infections, pain, and injuries throughout the body. 11
Table S8. ICPC-2 codes for physical disorders commonly encountered by PCPs 12
Table S9. ICPC-2 codes for mental disorders (excluding symptoms/complaints) 16
Table S10. Comparison of volume of PCP's mental-health encounters to encounters for common physical disorders grouped into 9 broad categories. 17
Figure S1. How does the number of PCP encounters for mental-health conditions compare to the volume of PCPs' encounters for common physical disorders grouped into 9 broad categories? 18
References 20

Table S1. International Classification of Primary Care (ICPC), 2nd edition. The table shows all the ICPC-2 chapter codes used in analyses reported in this article.

ICPC-2 – English

International Classification of Primary Care – 2nd Edition

Wonca International Classification Committee (WICC)

Wonca

Process codes

-30 Medical Exam/Eval-Complete
-31 Medical Examination/Health Evaluation-Partial/Pre-op check
-32 Sensitivity Test
-33 Microbiological/Immunological Test
-34 Blood Test
-35 Urine Test
-36 Faeces Test
-37 Histological/Exfoliative Cytology
-38 Other Laboratory Test NEC
-39 Physical Function Test
-40 Diagnostic Endoscopy
-41 Diagnostic Radiology/Imaging
-42 Electrical Tracings
-43 Other Diagnostic Procedures
-44 Preventive Imunisations/Medications
-45 Observe/Educate/Advice/Diet
-46 Consult with Primary Care Provider
-47 Consultation with Specialist
-48 Clarification/Discuss Patient's RFE
-49 Other Preventive Procedures
-50 Medicat-Script/Reqst/Renew/Inject
-51 Incise/Drain/Flush/Aspirate
-52 Excise/Remove/Biopsy/Destruction/Debride
-53 Instrument/Catheter/Intubate/Dilate
-54 Repair/Fixate-Suture/Cast/Prosthetic
-55 Local Injection/Infiltration
-56 Dress/Press/Compress/Tamponade
-57 Physical Medicine/Rehabilitation
-58 Therapeutic Counselling/Listening
-59 Other Therapeutic Procedure NEC
-60 Results Tests/Procedures
-61 Results Exam/Test/Record
-62 Administrative Procedure
-63 Follow-up Encounter Unspecified
-64 Encounter Initiated by Provider
-65 Encounter Initiated third person
-66 Refer to Other Provider (EXCL. M.D.)
-67 Referral to Physician/Specialist/Clinic/Hospital
-68 Other Referrals NEC
-69 Other Reason for Encounter NEC

General and Unspecified A

A01 Pain general/multiple sites
A02 Chills
A03 Fever
A04 Weakness/tiredness general
A05 Feeling ill
A06 Fainting/syncope
A07 Coma
A08 Swelling
A09 Sweating problem
A10 Bleeding/haemorrhage NOS
A11 Chest pain NOS
A13 Concern/fear medical treatment
A16 Irritable infant
A18 Concern about appearance
A20 Euthanasia request/discussion
A21 Risk factor for malignancy
A23 Risk factor NOS
A25 Fear of death/dying
A26 Fear of cancer NOS
A27 Fear of other disease NOS
A28 Limited function/disability NOS
A29 General symptom/complaint other
A70 Tuberculosis
A71 Measles
A72 Chickenpox
A73 Malaria
A74 Rubella
A75 Infectious mononucleosis
A76 Viral exanthem other
A77 Viral disease other/NOS
A78 Infectious disease other/NOS
A79 Malignancy NOS
A80 Trauma/injury NOS
A81 Multiple trauma/injuries
A82 Secondary effect of trauma
A84 Poisoning by medical agent
A85 Adverse effect medical agent
A86 Toxic effect non-medicinal substance
A87 Complication of medical treatment
A88 Adverse effect physical factor
A89 Effect prosthetic device
A90 Congenital anomaly OS/multiple
A91 Abnormal result investigation NOS
A92 Allergy/allergic reaction NOS
A93 Premature newborn
A94 Perinatal morbidity other
A95 Perinatal mortality
A96 Death
A97 No disease
A98 Health maintenance/prevention
A99 General disease NOS

Blood, Blood Forming Organs and Immune Mechanism B

B02 Lymph gland(s) enlarged/painful
B04 Blood symptom/complaint
B25 Fear of aids/HIV
B26 Fear cancer blood/lymph
B27 Fear blood/lymph disease other
B28 Limited function/disability
B29 Sympt/complt lymph/immune other
B70 Lymphadenitis acute
B71 Lymphadenitis non-specific
B72 Hodgkin's disease/lymphoma
B73 Leukaemia
B74 Malignant neoplasm blood other
B75 Benign/unspecified neoplasm blood
B76 Ruptured spleen traumatic
B77 Injury blood/lymph/spleen other
B78 Hereditary haemolytic anaemia
B79 Congen.anom. blood/lymph other
B80 Iron deficiency anaemia
B81 Anaemia, Vitamin B12/folate def.
B82 Anaemia other/unspecified
B83 Purpura/coagulation defect
B84 Unexplained abnormal white cells
B87 Splenomegaly
B90 HIV-infection/aids
B99 Blood/lymph/spleen disease other

PROCESS CODES
SYMPTOMS/COMPLAINTS
INFECTIONS
NEOPLASMS
INJURIES
CONGENITAL ANOMALIES
OTHER DIAGNOSES

Digestive D

D01 Abdominal pain/cramps general
D02 Abdominal pain epigastric
D03 Heartburn
D04 Rectal/anal pain
D05 Perianal itching
D06 Abdominal pain localized other
D07 Dyspepsia/indigestion
D08 Flatulence/gas/belching
D09 Nausea
D10 Vomiting
D11 Diarrhoea
D12 Constipation
D13 Jaundice
D14 Haematemesis/vomiting blood
D15 Melaena
D16 Rectal bleeding
D17 Incontinence of bowel
D18 Change faeces/bowel movements
D19 Teeth/gum symptom/complaint
D20 Mouth/tongue/lip symptom/complt.
D21 Swallowing problem
D23 Hepatomegaly
D24 Abdominal mass NOS
D25 Abdominal distension
D26 Fear of cancer of digestive system
D27 Fear of digestive disease other
D28 Limited function/disability (d)
D29 Digestive symptom/complaint other
D70 Gastrointestinal infection
D71 Mumps
D72 Viral hepatitis
D73 Gastroenteritis presumed infection
D74 Malignant neoplasm stomach
D75 Malignant neoplasm colon/rectum
D76 Malignant neoplasm pancreas
D77 Malig. neoplasm digest other/NOS
D78 Neoplasm digest benign/uncertain
D79 Foreign body digestive system
D80 Injury digestive system other
D81 Congen. anomaly digestive system
D82 Teeth/gum disease
D83 Mouth/tongue/lip disease
D84 Oesophagus disease
D85 Duodenal ulcer
D86 Peptic ulcer other
D87 Stomach function disorder
D88 Appendicitis
D89 Inguinal hernia
D90 Hiatus hernia
D91 Abdominal hernia other
D92 Diverticular disease
D93 Irritable bowel syndrome
D94 Chronic enteritis/ulcerative colitis
D95 Anal fissure/perianal abscess
D96 Worms/other parasites
D97 Liver disease NOS
D98 Cholecystitis/cholelithiasis
D99 Disease digestive system, other

Eye F

F01 Eye pain
F02 Red eye
F03 Eye discharge
F04 Visual floaters/spots
F05 Visual disturbance other
F13 Eye sensation abnormal
F14 Eye movements abnormal
F15 Eye appearance abnormal
F16 Eyelid symptom/complaint
F17 Glasses symptom/complaint
F18 Contact lens symptom/complaint
F27 Fear of eye disease
F28 Limited function/disability (f)
F29 Eye symptom/complaint other
F70 Conjunctivitis infectious
F71 Conjunctivitis allergic
F72 Blepharitis/stye/chalazion
F73 Eye infection/inflammation other
F74 Neoplasm of eye/adnexa
F75 Contusion/haemorrhage eye
F76 Foreign body in eye
F79 Injury eye other
F80 Blocked lacrimal duct of infant
F81 Congenital anomaly eye other
F82 Detached retina
F83 Retinopathy
F84 Macular degeneration
F85 Corneal ulcer
F86 Trachoma
F91 Refractive error
F92 Cataract
F93 Glaucoma
F94 Blindness
F95 Strabismus
F99 Eye/adnexa disease, other

Ear H

H01 Ear pain/earache
H02 Hearing complaint
H03 Tinnitus, ringing/buzzing ear
H04 Ear discharge
H05 Bleeding ear
H13 Plugged feeling ear
H15 Concern with appearance of ears
H27 Fear of ear disease
H28 Limited function/disability ear
H29 Ear symptom/complaint other
H70 Otitis externa
H71 Acute otitis media/myringitis
H72 Serous otitis media
H73 Eustachian salpingitis
H74 Chronic otitis media
H75 Neoplasm of ear
H76 Foreign body in ear
H77 Perforation ear drum
H78 Superficial injury of ear
H79 Ear injury other
H80 Congenital anomaly of ear
H81 Excessive ear wax
H82 Vertiginous syndrome
H83 Otosclerosis
H84 Presbyacusis
H85 Acoustic trauma
H86 Deafness
H99 Ear/mastoid disease, other

Cardiovascular K

K01 Heart pain
K02 Pressure/tightness of heart
K03 Cardiovascular pain NOS
K04 Palpitations/awareness of heart
K05 Irregular heartbeat other
K06 Prominent veins
K07 Swollen ankles/oedema
K22 Risk factor cardiovascular disease
K24 Fear of heart disease
K25 Fear of hypertension
K27 Fear cardiovascular disease other
K28 Limited function/disability (k)
K29 Cardiovascular sympt./compl. other
K70 Infection of circulatory system
K71 Rheumatic fever/heart disease
K72 Neoplasm cardiovascular
K73 Congenital anomaly cardiovascular
K74 Ischaemic heart disease w. angina
K75 Acute myocardial infarction
K76 Ischaemic heart disease w/o angina
K77 Heart failure
K78 Atrial fibrillation/flutter
K79 Paroxysmal tachycardia
K80 Cardiac arrhythmia NOS
K81 Heart/arterial murmur NOS
K82 Pulmonary heart disease
K83 Heart valve disease NOS
K84 Heart disease other
K85 Elevated blood pressure
K86 Hypertension uncomplicated
K87 Hypertension complicated
K88 Postural hypotension
K89 Transient cerebral ischaemia
K90 Stroke/cerebrovascular accident
K91 Cerebrovascular disease
K92 Atherosclerosis/PVD
K93 Pulmonary embolism
K94 Phlebitis/thrombophlebitis
K95 Varicose veins of leg
K96 Haemorrhoids
K99 Cardiovascular disease other

Musculoskeletal L

L01 Neck symptom/complain
L02 Back symptom/complaint
L03 Low back symptom/complaint
L04 Chest symptom/complaint
L05 Flank/axilla symptom/complaint
L07 Jaw symptom/complaint
L08 Shoulder symptom/complaint
L09 Arm symptom/complaint
L10 Elbow symptom/complaint
L11 Wrist symptom/complaint
L12 Hand/finger symptom/complaint
L13 Hip symptom/complaint
L14 Leg/thigh symptom/complaint
L15 Knee symptom/complaint
L16 Ankle symptom/complaint
L17 Foot/toe symptom/complaint
L18 Muscle pain
L19 Muscle symptom/complaint NOS
L20 Joint symptom/complaint NOS
L26 Fear of cancer musculoskeletal
L27 Fear musculoskeletal disease other
L28 Limited function/disability (l)
L29 Sympt/complt. Musculoskeletal other
L70 Infections musculoskeletal system
L71 Malignant neoplasm musculoskeletal
L72 Fracture: radius/ulna
L73 Fracture: tibia/fibula
L74 Fracture: hand/foot bone
L75 Fracture: femur
L76 Fracture: other
L77 Sprain/strain of ankle
L78 Sprain/strain of knee
L79 Sprain/strain of joint NOS
L80 Dislocation/subluxation
L81 Injury musculoskeletal NOS
L82 Congenital anomaly musculoskeletal
L83 Neck syndrome
L84 Back syndrome w/o radiating pain
L85 Acquired deformity of spine
L86 Back syndrome with radiating pain
L87 Bursitis/tendinitis/synovitis NOS
L88 Rheumatoid/seropositive arthritis
L89 Osteoarthrosis of hip
L90 Osteoarthrosis of knee
L91 Osteoarthrosis other
L92 Shoulder syndrome
L93 Tennis elbow
L94 Osteochondrosis
L95 Osteoporosis
L96 Acute internal damage knee
L97 Neoplasm benign/unspec musculo.
L98 Acquired deformity of limb
L99 Musculoskeletal disease, other

Neurological N

N01 Headache
N03 Pain face
N04 Restless legs
N05 Tingling fingers/feet/toes
N06 Sensation disturbance other
N07 Convulsion/seizure
N08 Abnormal involuntary movements
N16 Disturbance of smell/taste
N17 Vertigo/dizziness
N18 Paralysis/weakness
N19 Speech disorder
N26 Fear cancer neurological system
N27 Fear of neurological disease other
N28 Limited function/disability (n)
N29 Neurological symptom/complt. other
N70 Poliomyelitis
N71 Meningitis/encephalitis
N72 Tetanus
N73 Neurological infection other
N74 Malignant neoplasm nervous system
N75 Benign neoplasm nervous system
N76 Neoplasm nervous system unspec.
N79 Concussion
N80 Head injury other
N81 Injury nervous system other
N85 Congenital anomaly neurological
N86 Multiple sclerosis
N87 Parkinsonism
N88 Epilepsy
N89 Migraine
N90 Cluster headache
N91 Facial paralysis/bell's palsy
N92 Trigeminal neuralgia
N93 Carpal tunnel syndrome
N94 Peripheral neuritis/neuropathy
N95 Tension headache
N99 Neurological disease, other

Table S1. International Classification of Primary Care (ICPC), 2nd edition. (cont.)

Psychological P

P01 Feeling anxious/nervous/tense
P02 Acute stress reaction
P03 Feeling depressed
P04 Feeling/behaving irritable/angry
P05 Senility, feeling/behaving old
P06 Sleep disturbance
P07 Sexual desire reduced
P08 Sexual fulfilment reduced
P09 Sexual preference concern
P10 Stammering/stuttering/tic
P11 Eating problem in child
P12 Bedwetting/enuresis
P13 Encopresis/bowel training problem
P15 Chronic alcohol abuse
P16 Acute alcohol abuse
P17 Tobacco abuse
P18 Medication abuse
P19 Drug abuse
P20 Memory disturbance
P22 Child behaviour symptom/complaint
P23 Adolescent behav. Symptom/compl.
P24 Specific learning problem
P25 Phase of life problem adult
P27 Fear of mental disorder
P28 Limited function/disability (p)
P29 Psychological symptom/complt other
P70 Dementia
P71 Organic psychosis other
P72 Schizophrenia
P73 Affective psychosis
P74 Anxiety disorder/anxiety state
P75 Somatization disorder
P76 Depressive disorder
P77 Suicide/suicide attempt
P78 Neuraesthenia/surmenage
P79 Phobia/compulsive disorder
P80 Personality disorder
P81 Hyperkinetic disorder
P82 Post-traumatic stress disorder
P85 Mental retardation
P86 Anorexia nervosa/bulimia
P98 Psychosis NOS/other
P99 Psychological disorders, other

Respiratory R

R01 Pain respiratory system
R02 Shortness of breath/dyspnoea
R03 Wheezing
R04 Breathing problem, other
R05 Cough
R06 Nose bleed/epistaxis
R07 Sneezing/nasal congestion
R08 Nose symptom/complaint other
R09 Sinus symptom/complaint
R21 Throat symptom/complaint
R23 Voice symptom/complaint
R24 Haemoptysis
R25 Sputum/phlegm abnormal
R26 Fear of cancer respiratory system
R27 Fear of respiratory disease, other
R28 Limited function/disability (r)
R29 Respiratory symptom/complaint oth.
R71 Whooping cough
R72 Strep throat
R73 Boil/abscess nose
R74 Upper respiratory infection acute
R75 Sinusitis acute/chronic
R76 Tonsillitis acute
R77 Laryngitis/tracheitis acute
R78 Acute bronchitis/bronchiolitis
R79 Chronic bronchitis
R80 Influenza
R81 Pneumonia
R82 Pleurisy/pleural effusion
R83 Respiratory infection other
R84 Malignant neoplasm bronchus/lung
R85 Malinant neoplasm respiratory, other
R86 Benign neoplasm respiratory
R87 Foreign body nose/larynx/bronch
R88 Injury respiratory other
R89 Congenital anomaly respiratory
R90 Hypertrophy tonsils/adenoids
R92 Neoplasm respiratory unspecified
R95 Chronic obstructive pulmonary dis
R96 Asthma
R97 Allergic rhinitis
R98 Hyperventilation syndrome
R99 Respiratory disease other

PROCESS CODES
SYMPTOMS/COMPLAINTS
INFECTIONS
NEOPLASMS
INJURIES
CONGENITAL ANOMALIES
OTHER DIAGNOSES

Skin S

S01 Pain/tenderness of skin
S02 Pruritus
S03 Warts
S04 Lump/swelling localized
S05 Lumps/swellings generalized
S06 Rash localized
S07 Rash generalized
S08 Skin colour change
S09 Infected finger/toe
S10 Boil/carbuncle
S11 Skin infection post-traumatic
S12 Insect bite/sting
S13 Animal/human bite
S14 Burn/scald
S15 Foreign body in skin
S16 Bruise/contusion
S17 Abrasion/scratch/blister
S18 Laceration/cut
S19 Skin injury other
S20 Corn/callosity
S21 Skin texture symptom/complaint
S22 Nail symptom/complaint
S23 Hair loss/baldness
S24 Hair/scalp symptom/complaint
S26 Fear of cancer of skin
S27 Fear of skin disease other
S28 Limited function/disability (s)
S29 Skin symptom/complaint other
S70 Herpes zoster
S71 Herpes simplex
S72 Scabies/other acariasis
S73 Pediculosis/skin infestation other
S74 Dermatophytosis
S75 Moniliasis/candidiasis skin
S76 Skin infection other
S77 Malignant neoplasm of skin
S78 Lipoma
S79 Neoplasm skin benign/unspecified
S80 Solar keratosis/sunburn
S81 Haemangioma/lymphangioma
S82 Naevus/mole
S83 Congenital skin anomaly other
S84 Impetigo
S85 Pilonidal cyst/fistula
S86 Dermatitis seborrhoeic
S87 Dermatitis/atopic eczema
S88 Dermatitis contact/allergic
S89 Diaper rash
S90 Pityriasis rosea
S91 Psoriasis
S92 Sweat gland disease
S93 Sebaceous cyst
S94 Ingrowing nail
S95 Molluscum contagiosum
S96 Acne
S97 Chronic ulcer skin
S98 Urticaria
S99 Skin disease, other

Endocrine/Metabolic and Nutritional T

T01 Excessive thirst
T02 Excessive appetite
T03 Loss of appetite
T04 Feeding problem of infant/child
T05 Feeding problem of adult
T07 Weight gain
T08 Weight loss
T10 Growth delay
T11 Dehydration
T26 Fear of cancer of endocrine system
T27 Fear endocrine/metabolic dis other
T28 Limited function/disability (t)
T29 Endocrine/met./sympt/complt other
T70 Endocrine infection
T71 Malignant neoplasm thyroid
T72 Benign neoplasm thyroid
T73 Neoplasm endocrine oth/unspecified
T78 Thyroglossal duct/cyst
T80 Congenital anom endocrine/metab.
T81 Goitre
T82 Obesity
T83 Overweight
T85 Hyperthyroidism/thyrotoxicosis
T86 Hypothyroidism/myxoedema
T87 Hypoglycaemia
T89 Diabetes insulin dependent
T90 Diabetes non-insulin dependent
T91 Vitamin/nutritional deficiency
T92 Gout
T93 Lipid disorder
T99 Endocrine/metab/nutrit. dis. other

Urological U

U01 Dysuria/painful urination
U02 Urinary frequency/urgency
U04 Incontinence urine
U05 Urination problems other
U06 Haematuria
U07 Urine symptom/complaint other
U08 Urinary retention
U13 Bladder symptom/complaint other
U14 Kidney symptom/complaint
U26 Fear of cancer of urinary system
U27 Fear of urinary disease other
U28 Limited function/disability urinary
U29 Urinary symptom/complaint other
U70 Pyelonephritis/pyelitis
U71 Cystitis/urinary infection other
U72 Urethritis
U75 Malignant neoplasm of kidney
U76 Malignant neoplasm of bladder
U77 Malignant neoplasm urinary other
U78 Benign neoplasm urinary tract
U79 Neoplasm urinary tract NOS
U80 Injury urinary tract
U85 Congenital anomaly urinary tract
U88 Glomerulonephritis/nephrosis
U90 Orthostatic albumin./proteinuria
U95 Urinary calculus
U98 Abnormal urine test NOS
U99 Urinary disease, other

Pregnancy, Childbearing, Family Planning W

W01 Question of pregnancy
W02 Fear of pregnancy
W03 Antepartum bleeding
W05 Pregnancy vomiting/nausea
W10 Contraception postcoital
W11 Contraception oral
W12 Contraception intrauterine
W13 Sterilization
W14 Contraception other
W15 Infertility/subfertility
W17 Post-partum bleeding
W18 Post-partum symptom/complaint oth.
W19 Breast/lactation symptom/complaint
W21 Concern body image in pregnancy
W27 Fear complications of pregnancy
W28 Limited function/disability (w)
W29 Pregnancy symptom/complaint other
W70 Puerperal infection/sepsis
W71 Infection complicating pregnancy
W72 Malignant neoplasm relate to preg.
W73 Benign/unspec. neoplasm/pregnancy
W75 Injury complicating pregnancy
W76 Congenital anomaly complicate preg.
W78 Pregnancy
W79 Unwanted pregnancy
W80 Ectopic pregnancy
W81 Toxaemia of pregnancy
W82 Abortion spontaneous
W83 Abortion induced
W84 Pregnancy high risk
W85 Gestational diabetes
W90 Uncomplicate labour/delivery live
W91 Uncomplicate labour/delivery still
W92 Complicate labour/ delivery livebirth
W93 Complicate labour/delivery stillbirth
W94 Puerperal mastitis
W95 Breast disorder in pregnancy other
W96 Complications of puerperium other
W99 Disorder pregnancy/delivery, other

Female Genital X

X01 Genital pain female
X02 Menstrual pain
X03 Intermenstrual pain
X04 Painful intercourse female
X05 Menstruation absent/scanty
X06 Menstruation excessive
X07 Menstruation irregular/frequent
X08 Intermenstrual bleeding
X09 Premenstrual symptom/complaint
X10 Postponement of menstruation
X11 Menopausal symptom/complaint
X12 Postmenopausal bleeding
X13 Postcoital bleeding
X14 Vaginal discharge
X15 Vaginal symptom/complaint other
X16 Vulval symptom/complaint
X17 Pelvis symptom/complaint female
X18 Breast pain female
X19 Breast lump/mass female
X20 Nipple symptom/complaint female
X21 Breast symptom/complt. female other
X22 Concern breast appearance female
X23 Fear sexually transmitted disease (f)
X24 Fear of sexual dysfunction female
X25 Fear of genital cancer female
X26 Fear of breast cancer female
X27 Fear genital/breast disease other (f)
X28 Limited function/disability (x)
X29 Genital symptom/complt female oth.
X70 Syphilis female
X71 Gonorrhoea female
X72 Genital candidiasis female
X73 Genital trichomoniasis female
X74 Pelvic inflammatory disease
X75 Malignant neoplasm cervix
X76 Malignant neoplasm breast female
X77 Malignant neoplasm genital other (f)
X78 Fibromyoma uterus
X79 Benign neoplasm breast female
X80 Benign neoplasm female genital
X81 Genital neoplasm oth/unspecied (f)
X82 Injury genital female
X83 Congenital anomaly genital female
X84 Vaginitis/vulvitis NOS
X85 Cervical disease NOS
X86 Abnormal cervix smear
X87 Uterovaginal prolapse
X88 Fibrocystic disease breast
X89 Premenstrual tension syndrome
X90 Genital herpes female
X91 Condylomata acuminata female
X92 Chlamydia infection genital (f)
X99 Genital disease female, other

Male Genital Y

Y01 Pain in penis
Y02 Pain in testis/scrotum
Y03 Urethral discharge
Y04 Penis symptom/complaint other
Y05 Scrotum/testis sympt/complt. other
Y06 Prostate symptom/complaint
Y07 Impotence NOS
Y08 Sexual function sympt./complt.(m)
Y10 Infertility/subfertility male
Y13 Sterilization male
Y14 Family planning male other
Y16 Breast symptom/complaint male
Y24 Fear of sexual dysfunction male
Y25 Fear sexually transmitted dis. male
Y26 Fear of qenital cancer male
Y27 Fear of genital disease male other
Y28 Limited function/disability (y)
Y29 Genital sympt./complt.male other
Y70 Syphilis male
Y71 Gonorrhoea male
Y72 Genital herpes male
Y73 Prostatitis/seminal vesiculitis
Y74 Orchitis/epididymitis
Y75 Balanitis
Y76 Condylomata acuminata male
Y77 Malignant neoplasm prostate
Y78 Malign neoplasm male genital other
Y79 Benign/unspec. neoplasm gen. (m)
Y80 Injury male genital
Y81 Phimosis/redundant prepuce
Y82 Hypospadias
Y83 Undescended testicle
Y84 Congenital genl anomaly (m) other
Y85 Benign prostatic hypertrophy
Y86 Hydrocoele
Y99 Genital disease male, other

Social Problems Z

Z01 Poverty/financial problem
Z02 Food/water problem
Z03 Housing/neighbourhood problem
Z04 Social cultural problem
Z05 Work problem
Z06 Unemployment problem
Z07 Education problem
Z08 Social welfare problem
Z09 Legal problem
Z10 Health care system problem
Z11 Compliance/being ill problem
Z12 Relationship problem with partner
Z13 Partner's behaviour problem
Z14 Partner illness problem
Z15 Loss/death of partner problem
Z16 Relationship problem with child
Z18 Illness problem with child
Z19 Loss/death of child problem
Z20 Relationship prob. parent/family
Z21 Behaviour problem parent/family
Z22 Illness problem parent/family
Z23 Loss/death parent/family member
Z24 Relationship problem friend
Z25 Assault/harmful event problem
Z27 Fear of a social problem
Z28 Limited function/disability (z)
Z29 Social problem NOS

Abbreviations

Anom	anomaly
behav.	behaviour
bronch.	bronchus
complicat.	complication
congen.	congenital
dis.	disease
eval.	evaluation
exam.	examination
gen.	genital
malig.	malignant
metab.	metabolic
musculo.	musculoskeletal
NEC	not elsewhere classified
NOS	not otherwise specified
nutrit.	nutrition
oth	other
preg.	pregnancy
prob.	problem
RFE	reason for encounter
sympt.	symptom
unspec.	unspecified
w	with
w/o	without

Table S2. ICPC-2 codes for mental-health conditions. We grouped mental-health conditions into 24 categories. The list of codes is presented on this table.

Mental Health Category	Code	Code description
Acute Stress Reaction	P02	Acute stress reaction
ADHD	P81	Hyperkenetic disorder
Anxiety	P01	Feeling anxious/nervous/tense
	P74	Anxiety disorder/anxiety state
Child/adolescent behavior symptoms/complaints	P22	Child behaviour symptom/complaint
	P23	Adolescent behavior symptom/complaint
Chronic fatigue	P78	Neuraesthenia/surmenage
Continence issues	P12	Bedwetting/enuresis
	P13	Encopresis/bowel training problem
Dementia/Memory disturbance	P05	Senility, feeling/behavior old
	P20	Memory disturbance
	P70	Dementia
Depression	P03	Feeling depressed
	P76	Depressive disorder
Developmental delay/learning problems	P24	Specific learning problem
	P28	Limited function/disability (p)
	P85	Mental retardation
Eating Disorder	P11	Eating problem in child
	P86	Anorexia nervosa/bulimia
Fear of mental disorder	P27	Fear of mental disorder
Feeling/behaving irritable/angry	P04	Feeling/behaving irritable/angry
Personality disorder	P80	Personality disorder
Phase of life problem in adult	P25	Phase of life problem adult
Phobia/compulsive disorder	P79	Phobia/compulsive disorder
Psychosis	P71	Organic psychosis other
	P72	Schizophrenia
	P73	Affective psychosis
	P98	Psychosis NOS/other
PTSD	P82	Post-traumatic stress disorder
Sexual concerns	P07	Sexual desire reduced
	P08	Sexual fulfilment reduced
	P09	Sexual preference concern
Sleep Disturbance	P06	Sleep disturbance
Somatization	P75	Somatization disorder
Stammering/stuttering/tic	P10	Stammering/stuttering/tic
Substance Abuse	P15	Chronic alcohol abuse
	P16	Acute alcohol abuse
	P17	Tobacco abuse
	P18	Medication abuse
	P19	Drug abuse

Table S2. ICPC-2 codes for mental-health conditions. (cont)

Mental Health Category	Code	Code description
Suicide/Suicide attempt	P77	Suicide/suicide attempt
"Other" psychological symptom/disease	P29	Psychological symptom/complaint other
	P99	Psychological disorders, other

Table S3. ICPC-2 codes for infections, pain, and injuries. Because infections, pain, and injuries are distributed throughout different chapters in the ICPC-2 according to the body system they involve, we pooled the relevant codes across the different chapters. The list of codes is presented in this table.

Infection Codes			
A70	Tuberculosis	R79	Chronic bronchitis
A71	Measles	R80	Influenza
A72	Chickenpox	R81	Pneumonia
A73	Malaria	R82	Pleurisy/pleural effusion
A74	Rubella	R83	Respiratory infection other
A75	Infectious mononucleosis	S03	Warts
A76	Viral exanthem other	S09	Infected finger/toe
A77	Viral disease other/NOS	S10	Boil/carbuncle
A78	Infectious disease other/NOS	S11	Skin infection post-traumatic
B70	Lymphadenitis acute	S70	Herpes zoster
B71	Lymphadenitis non-specific	S71	Herpes simplex
D70	Gastrointestinal infection	S72	Scabies/other acariasis
D71	Mumps	S73	Pediculosis/skin infestation other
D72	Viral hepatitis	S74	Dermatophytosis
D73	Gastroenteritis presumed infection	S75	Moniliasis/candidiasis skin
F70	Conjunctivitis infectious	S76	Skin infection other
F71	Conjunctivitis allergic	S84	Impetigo
F72	Blepharitis/stye/chalazion	S95	Molluscum contagiosum
F73	Eye infection/inflammation other	T70	Endocrine infection
H70	Otitis externa	U70	Pyelonephritis/pyelitis
H71	Acute otitis media/myringitis	U71	Cystitis/urinary infection other
H72	Serious otitis media	U72	Urethritis
H73	Eustachian salpingitis	W70	Puerperal infection/sepsis
H74	Chronic otitis media	W71	Infection complicating pregnancy
K70	Infection of circulatory system	X70	Syphilis female
K71	Rheumatic fever/hear disease	X71	Gonorrhoea female
L70	Infections musculoskeletal system	X72	Genital candidiasis female
N70	Poliomyelitis	X73	Genital trichomoniasis female
N71	Meningitis/encephalitis	X74	Pelvic inflammatory disease
N72	Tetanus	X90	Genital herpes female
N73	Neurological infection other	X91	Condylomata acuminata female
R71	Whopping cough	X92	Chlamydia infection genital (f)
R72	Strep throat	Y70	Syphilis male
R73	Boil/abscess nose	Y71	Gonorrhoea male
R74	Upper respiratory infection acute	Y72	Genital herpes male
R75	Sinusitis acute/chronic	Y73	Prostatitis/seminal vesiculitis
R76	Tonsillitis acute	Y74	Orchitis/epididymitis
R77	Laryngitis/tracheitis acute	Y75	Balanitis
R78	Acute bronchitis/bronchiolitis	Y76	Condylomata acuminata male

Table S3. ICPC-2 codes for infections, pain, and injuries. (cont.)

**Pain Codes**			
A01	Pain general/multiple sites	L86	Back syndrome with radiating pain
A11	Chest pain NOS	N03	Pain face
B02	Lymph gland(s) enlarged/painful	R01	Pain respiratory system
D01	Abdominal pain/cramps general	S01	Pain/tenderness of skin
D02	Abdominal pain epigastric	U01	Dysuria/painful urination
D04	Rectal/anal pain	X01	Genital pain female
D06	Abdominal pain localized other	X02	Menstrual pain
F01	Eye pain	X03	Intermenstrual pain
H01	Ear pain/earache	X04	Painful intercourse female
K01	Heart pain	X18	Breast pain female
K03	Cardiovascular pain NOS	Y01	Pain in penis
L18	Muscle pain	Y02	Pain in testis/scrotum
**Injury Codes**			
A80	Trauma/injury NOS	L76	Fracture: other
A81	Multiple trauma/injuries	L77	Sprain/strain of ankle
A82	Secondary effect of trauma	L78	Sprain/strain of knee
A84	Poisoning by a medical agent	L79	Sprain/strain of join NOS
A85	Adverse effect medical agent	L80	Dislocation/subluxation
A86	Toxic effect non-medicinal substance	L81	Injury musculoskeletal NOS
A87	Complication of medical treatment	L96	Acute internal damage knee
A88	Adverse effect physical factor	N79	Concussion
A89	Effect prosthetic device	N80	Head injury other
B76	Ruptured spleen traumatic	N81	Injury nervous system other
B77	Injury blood/lymph/spleen other	R87	Foreign body nose/larynx/bronch
D79	Foreign body digestive system	R88	Injury respiratory other
D80	Injury digestive system other	S12	Insect bite/sting
F75	Contusion/haemorrhage eye	S13	Animal/human bite
F76	Foreign body in eye	S14	Burn/scald
F79	Injury eye other	S15	Foreign body in skin
H76	Foreign body in ear	S16	Bruise/contusion
H77	Perforation ear drum	S17	Abrasion/scratch/blister
H78	Superficial injury of ear	S18	Laceration/cut
H79	Ear injury other	S19	Skin injury other
L72	Fracture: radius/ulna	U80	Injury urinary tract
L73	Fracture: tibia/fibula	W75	Injury complicating pregnancy
L74	Fracture: hand/foot bone	X82	Injury genital female
L75	Fracture: femur	Y80	Injury male genital

Table S4. Proportion of patients presenting with different mental-health conditions.

	Everyone		Females		Males	
Mental Health Condition	N	%	N	%	N	%
Any Psychological	2,309,787	47.4	1,285,868	52.7	1,023,919	42.1
Depression	897,220	18.4	559,560	22.9	337,660	13.9
Sleep disturbance	764,955	15.7	447,280	18.3	317,675	13.1
Acute stress reaction	614,659	12.6	401,612	16.4	213,047	8.8
Anxiety	509,558	10.5	324,992	13.3	184,566	7.6
Dementia/Memory problems	272,134	5.6	154,130	6.3	118,004	4.8
Substance abuse	231,307	4.7	92,684	3.8	138,623	5.7
Psychosis	112,683	2.3	59,244	2.4	53,439	2.2
ADHD	109,162	2.2	41,403	1.7	67,759	2.8
Phobia/Compulsive disorder	102,645	2.1	58,142	2.4	44,503	1.8
Sexual concern	88,901	1.8	4,660	0.2	84,241	3.5
Child/Adolescent behavior symptom/complaint	81,810	1.7	31,695	1.3	50,115	2.1
Developmental delay/Learning problems	64,987	1.3	28,240	1.2	36,747	1.5
PTSD	51,130	1.0	36,511	1.5	14,619	0.6
Personality disorder	43,580	0.9	22,022	0.9	21,558	0.9
Continence issues	41,541	0.9	14,138	0.6	27,403	1.1
Suicide/Suicide attempt	40,052	0.8	19,023	0.8	21,029	0.9
Neuresthenia/surmenage (chronic fatigue)	37,700	0.8	26,139	1.1	11,561	0.5
Somatization	34,953	0.7	22,825	0.9	12,128	0.5
Fear of mental disorder	34,398	0.7	16,949	0.7	17,449	0.7
Eating disorder	20,828	0.4	16,934	0.7	3,894	0.2
Phase of life problem adult	20,333	0.4	13,043	0.5	7,290	0.3
Stammering/stuttering/tic	17,614	0.4	4,907	0.2	12,707	0.5
Feeling/behaving irritable/angry	17,290	0.4	8,138	0.3	9,152	0.4
Other psychological symptom/disease	591,003	12.1	367,135	15.0	223,868	9.2

Table S5. Proportion of mental-health encounters devoted to different mental-health conditions.

	All		Females		Males	
Mental Health Condition	N	%	N	%	N	%
Any Psychological	41,616,704	11.7	24,949,262	12.0	16,667,442	11.4
Depression	9,898,619	23.8	6,547,697	26.2	3,350,922	20.1
Anxiety	5,854,719	14.1	3,823,424	15.3	2,031,295	12.2
Sleep disturbance	5,051,797	12.1	3,232,751	13.0	1,819,046	10.9
Substance abuse	3,448,489	8.3	1,140,702	4.6	2,307,787	13.8
Acute stress reaction	2,935,283	7.1	2,057,670	8.2	877,613	5.3
Psychosis	2,858,959	6.9	1,565,787	6.3	1,293,172	7.8
Dementia/Memory problems	2,228,914	5.4	1,387,209	5.6	841,705	5.0
ADHD	1,598,575	3.8	606,325	2.4	992,250	6.0
Phobia/Compulsive disorder	703,696	1.7	373,248	1.5	330,448	2.0
Developmental delay/Learning problems	617,385	1.5	295,582	1.2	321,803	1.9
PTSD	525,608	1.3	388,780	1.6	136,828	0.8
Personality disorder	452,721	1.1	288,548	1.2	164,173	1.0
Child/Adolescent behavior symptom/complaint	285,076	0.7	108,342	0.4	176,734	1.1
Sexual concern	266,548	0.6	13,645	0.1	252,903	1.5
Neuresthenia/surmenage (chronic fatigue)	226,095	0.5	163,540	0.7	62,555	0.4
Eating disorder	185,880	0.4	171,674	0.7	14,206	0.1
Somatization	176,970	0.4	126,790	0.5	50,180	0.3
Continence issues	132,817	0.3	43,079	0.2	89,738	0.5
Suicide/Suicide attempt	94,090	0.2	49,442	0.2	44,648	0.3
Phase of life problem adult	82,109	0.2	56,436	0.2	25,673	0.2
Stammering/stuttering/tic	75,152	0.2	19,787	0.1	55,365	0.3
Fear of mental disorder	72,765	0.2	36,058	0.1	36,707	0.2
Feeling/behaving irritable/angry	40,023	0.1	18,917	0.1	21,106	0.1
Other psychological symptom/disease	3,804,414	9.1	2,433,829	9.8	1,370,585	8.2

Note: Percentages for “Any Psychocological” are based on the total number encounters across all body systems; all other percentages are based on the total number of mental health encounters.

Table S6. Comparison of the volume of PCP's mental-health encounters to encounters for medical conditions in 15 different body systems.

ICPC-2 Chapter	Body System	All		Females		Males	
		N	%	N	%	N	%
P	Psychological	41,616,704	11.7	24,949,262	12.0	16,667,442	11.4
L	Musculoskeletal	61,516,933	17.4	37,377,893	18.0	24,139,040	16.5
K	Cardiovascular	42,926,196	12.1	20,866,369	10.0	22,059,827	15.1
R	Respiratory	38,917,254	11.0	21,808,555	10.5	17,108,699	11.7
A	General and Unspecified	36,372,091	10.3	21,061,164	10.1	15,310,927	10.5
S	Skin	23,559,554	6.6	12,850,868	6.2	10,708,686	7.3
T	Endocrine/Metabolic and Nutritional	23,483,464	6.6	13,772,062	6.6	9,711,402	6.6
D	Digestive	21,400,200	6.0	12,576,461	6.0	8,823,739	6.0
N	Neurological	13,633,579	3.8	8,490,696	4.1	5,142,883	3.5
U	Urological	12,211,775	3.4	8,118,761	3.9	4,093,014	2.8
X	Male/Female Genital	10,886,246	3.1	6,343,291	3.0	4,542,955	3.1
W	Pregnancy, Childbearing, Family Planning	10,179,018	2.9	10,026,994	4.8	152,024	0.1
F	Eye	6,942,772	2.0	3,840,146	1.8	3,102,626	2.1
H	Ear	6,431,506	1.8	3,386,761	1.6	3,044,745	2.1
B	Blood, Blood Forming Organs and Immune Function	4,438,999	1.3	2,614,672	1.3	1,824,327	1.2

Table S7. Comparison of the volume of PCP's mental-health encounters to encounters for infections, pain, and injuries throughout the body.

	All		Females		Males	
Health Category	N	%	N	%	N	%
Psychological	41,616,704	11.7	24,949,262	12.0	16,667,442	11.4
Infection	40,630,950	11.5	24,215,997	11.6	16,414,953	11.2
Pain	16,993,513	4.8	11,007,248	5.3	5,986,265	4.1
Injury	13,626,383	3.8	6,647,956	3.2	6,978,427	4.8

Table S8. ICPC-2 codes for physical disorders commonly encountered by PCPs. We grouped 31 disorders grouped within nine broad categories: circulatory, endocrine, pulmonary, gastrointestinal, urogenital, musculoskeletal, hematologic, and neurologic conditions and cancer. We report these 31 disorders grouped into nine categories because this coding system has been used in previous research about the connection between mental disorders and physical disorders (Momen 2020). Because previous research used ICD-10 codes from hospitalization and prescription data, we matched the 31 ICD-10 disorders to ICPC-2 disorder codes with the aid of the Norwegian Directorate of eHealth's ICPC-2 to ICD-10 mapping (https://www.ehelse.no/kodeverk-og-terminologi/ICPC-2/icpc-2e-english-version). The list of codes and the cross-walk are presented in this table.

**Category/Condition (from Momen et al., 2020 unless otherwise specified)**	**Momen ICD-10 Codes**	**ICPC-2 Code**	**ICPC-2 Condition**
**Circulatory System**			
Hypertension	I10	K86	Hypertension uncomplicated
	I11-I13, I15	K87	Hypertension complicated
Dyslipidemia	E78	T93	Lipid disorder
Ischemic heart disease	I20, I24	K74	Ischaemic heart disease with angina
	I21-I24	K75	Acute myocardial infarction
	I25	K76	Ischaemic heart disease without angina
Atrial fibrilation	I48	K78	Atrial fibrillation/flutter
Heart failure	I50	K77	Heart failure
Peripheral artery occlusive disease	I70, I73-I74	K92	Atherosclerosis/peripheral vascular disease
Stroke	I60-I64	K90	Stroke/cerebrovascular accident
	I69	K91	Cerebrovascular disease
Valvular disease (Launders et al., 2022)	I34-I37, I39	K83	Heart valve disease NOS
Pulmonary circulatory disease (Launders et al., 2022)	I27-I28	K82	Pulmonary heart disease
Cardiac arrhythmia (Launders et al., 2022)	I49	K80	Cardiac arrhythmia NOS

Table S8. ICPC-2 codes for physical disorders commonly encountered by PCPs (cont.).

Category/Condition (from Momen et al., 2020 unless otherwise specified)	Momen ICD-10 Codes	ICPC-2 Code	ICPC-2 Condition
**Endocrine System**			
Diabetes mellitus	E10	T89	Diabetes insulin dependent
	E11-E14	T90	Diabetes non-insulin dependent
Thyroid disorder	E00	T80	Congenital anomaly endocrine/metabolic
	E01-E03	T86	Hypothyroidism/myxoedema
	E04	T81	Goitre
	E05	T85	Hyperthyroidism/thyrotoxicosis
Gout	M10	T92	Gout
**Pulmonary System and Allergy**			
Chronic pulmonary disease	J40	R78	Acute bronchitis/bronchiolitis
	J41-J42	R79	Chronic bronchitis
	J43-J44	R95	Chronic obstructive pulmonary disease
	J45-J46	R96	Asthma
Allergy	J30	R97	Allergic rhinitis
	L23	S88	Dermatitis contact/allergic
	T78.0, T78.2, T78.4	A92	Allergy/allergic reaction NOS
**Gastrointestinal System**			
Ulcer/chronic gastritis	K25, K27-K28	D86	Peptic ulcer other
	K26	D85	Duodenal ulcer
	K29.3, K29.5	D87	Stomach function disorder
Chronic liver disease	B16-B19	D72	Viral hepatitis
	K70, K74, K76.6	D97	Liver disease NOS
Inflammatory bowel disease	K50, K51	D94	Chronic enteritis/ulcerative colitis
Diverticular disease of intestine	K57	D92	Diverticular disease
**Urogenital System**			
Chronic kidney disease	N03	U88	Glomerulonephritis/nephrosis
	N11	U70	Pyelonephritis/pyelitis
Prostate disorders	N40	Y85	Benign prostatic hypertrophy

Table S8. **ICPC-2 codes for physical disorders commonly encountered by PCPs (cont.)**.

Category/Condition (from Momen et al., 2020 unless otherwise specified)	Momen ICD-10 Codes	ICPC-2 Code	ICPC-2 Condition
**Musculoskeletal System**			
Connective tissue disorders	M05-M06, M08	L88	Rheumatoid/seropositive arthritis
Osteoporosis	M80-M82	L95	Osteoporosis
**Hematological System**			
HIV/AIDS	B20-B24	B90	HIV-infection/AIDS
Anemias	D50	B80	Iron deficiency anaemia
	D51-D52	B81	Anaemia vit B12/folate deficiency
	D53, D55, D59-D61, D63-D64	B82	Anaemia other/unspecified
	D56-D58	B78	Hereditary haemolytic anaemia
Coagulopathy (Launders et al., 2022)	D65-D69	B83	Purpura/coagulation defect
**Cancers**			
	C00-C08, C14-C15, C17, C22-C24, C26, C45, C48	D77	Malignant digestive neoplasm other/NOS
	C09-C14, C31-C32, C38-C39, C45	R85	Malignant neoplasm respiratory other
	C16	D74	Malignant neoplasm stomach
	C18-C21	D75	Malignant neoplasm colon/rectum
	C25	D76	Malignant neoplasm pancreas
	C26, C37, C77, C88, C90, C94, C96	B74	Malignant neoplasm blood other
	C30, C49	H75	Neoplasm of ear
	C33-C34, C45	R84	Malignant neoplasm bronchus/lung
	C38, C45, C76, C78-C80, C97	A79	Malignancy NOS
	C38, C45	K72	Neoplasm cardiovascular
	C40-C41, C49	L71	Malignant neoplasm musculoskeletal
	C43, C46	S77	Malignant neoplasm of skin
	C47, C70-C72	N74	Malignant neoplasm nervous system
	C50	X76	Malignant neoplasm breast female
	C50, C60, C62-C63	Y78	Malignant neoplasm male genital other
	C51, C54-C57	X77	Malignant neoplasm genital female other

Table S8. ICPC-2 codes for physical disorders commonly encountered by PCPs (cont.).

Category/Condition (from Momen et al., 2020 unless otherwise specified)	Momen ICD-10 Codes	ICPC-2 Code	ICPC-2 Condition
	**Cancers, cont.**		
	C53	X75	Malignant neoplasm cervix
	C58	W72	Malignant neoplasm related to pregnancy
	C61	Y77	Malignant neoplasm prostate
	C64-C65	U75	Malignant neoplasm of kidney
	C66, C68	U77	Malignant neoplasm urinary tract other
	C67	U76	Malignant neoplasm of bladder
	C69	F74	Neoplasm of eye/adnexa
	C73	T71	Malignant neoplasm thyroid
	C74	T73	Neoplasm endocrine other/unspecified
	C81-C86	B72	Hodgkin Lymphoma
	C91-C95	B73	Leukaemia
	**Neurological System**		
Migraine	G43	N89	Migraine
Epilepsy	G40-G41	N88	Epilepsy
Parkinson's disease	G20-G22	N87	Parkinsonism
Multiple schlerosis	G35	N86	Multiple sclerosis
Neuropathies	G50	N92	Trigeminal neuralgia
	G51, G53	N91	Facial paralysis/bell's palsy
	G54-G64	N94	Peripheral neuritis/neuropathy
	G55	L86	Back syndrome with radiating pain
	G56	N93	Carpal tunnel syndrome
Paralysis or paresis (Launders et al., 2022)	G81-G83, G98	N18	Paralysis/weakness

Table S9. ICPC-2 codes for mental disorders (excluding symptoms/complaints). We restricted mental-health conditions to codes that define mental disorders. These are ICPC-2 Chapter P codes > 70 as well as codes for substance abuse. The list of codes is presented in this table.

P15	Chronic alcohol abuse
P17	Tobacco abuse
P18	Medication abuse
P19	Drug abuse
P70	Dementia
P71	Organic psychosis other
P72	Schizophrenia
P73	Affective psychosis
P74	Anxiety disorder/anxiety state
P75	Somatization disorder
P76	Depressive disorder
P77	Suicide/suicide attempt
P78	Neuraesthenia/surmenage
P79	Phobia/compulsive disorder
P80	Personality disorder
P81	Hyperkenetic disorder
P82	Post-traumatic stress disorder
P85	Mental retardation
P86	Anorexia nervosa/bulimia
P98	Psychosis NOS/other
P99	Psychological disorders, other

Table S10. Comparison of volume of PCP's mental-health encounters to encounters for common physical disorders grouped into 9 broad categories.

	All		Females		Males	
Health Category	N	%	N	%	N	%
Psychological	41,616,704	11.7	24,949,262	12.0	16,667,442	11.4
Psychological (excluding symptoms/complaints)	25,023,819	7.1	14,329,504	6.9	10,694,315	7.3
Circulatory System	35,596,783	10.0	17,110,046	8.2	18,486,737	12.6
Pulmonary System and Allergy	14,649,721	4.1	8,086,624	3.9	6,563,097	4.5
Endocrine System	13,793,868	3.9	7,803,875	3.8	5,989,993	4.1
Neurological System	8,813,449	2.5	5,218,185	2.5	3,595,264	2.5
Cancers	6,496,273	1.8	3,023,778	1.5	3,472,495	2.4
Musculoskeletal System	3,941,251	1.1	2,933,486	1.4	1,007,765	0.7
Hematological System	2,586,796	0.7	1,646,711	0.8	940,085	0.6
Gastrointestinal System	2,326,786	0.7	1,320,895	0.6	1,005,891	0.7
Urogenital System	920,323	0.3	228,575	0.1	691,748	0.5

Figure S1. How does the number of PCP encounters for mental-health conditions compare to the volume of PCPs' encounters for common physical disorders grouped into 9 broad categories?

We examined 31 disorders grouped within nine broad categories: circulatory, endocrine, pulmonary, gastrointestinal, urogenital, musculoskeletal, hematologic, and neurologic conditions and cancer. We chose these 31 disorders grouped into nine categories because this coding system has been used in previous research about the connection between mental disorders and physical disorders (Momen 2020). Because previous research used ICD-10 codes from hospitalization and prescription data, we mapped the 31 ICD-10 disorders to ICPC-2 disorder codes using the Norwegian Directorate of eHealth's ICPC-2-to-ICD-10 tool (https://www.ehelse.no/kodeverk-og-terminologi/ICPC-2/icpc-2e-english-version) (**Table S8)**.

The figure shows that PCPs had more mental-health encounters than they did encounters for physical disorders ranging from circulatory to urogenital disorders. This comparison may not be fair, because it compares diagnosed physical disorders to diagnoses *and also* symptoms/complaints of mental-health conditions. However, the figure shows that even when we excluded symptoms/complaints to restrict mental-health conditions to diagnostic codes that define mental disorders (**Table S9**), encounters for mental disorders were more frequent than encounters for eight other broad categories of physical diseases, excepting only circulatory disorders (**Table S10**).

Figure S1. How does the number of PCP encounters for mental-health conditions compare to the volume of PCPs' encounters for common physical disorders grouped into 9 broad categories? (cont).

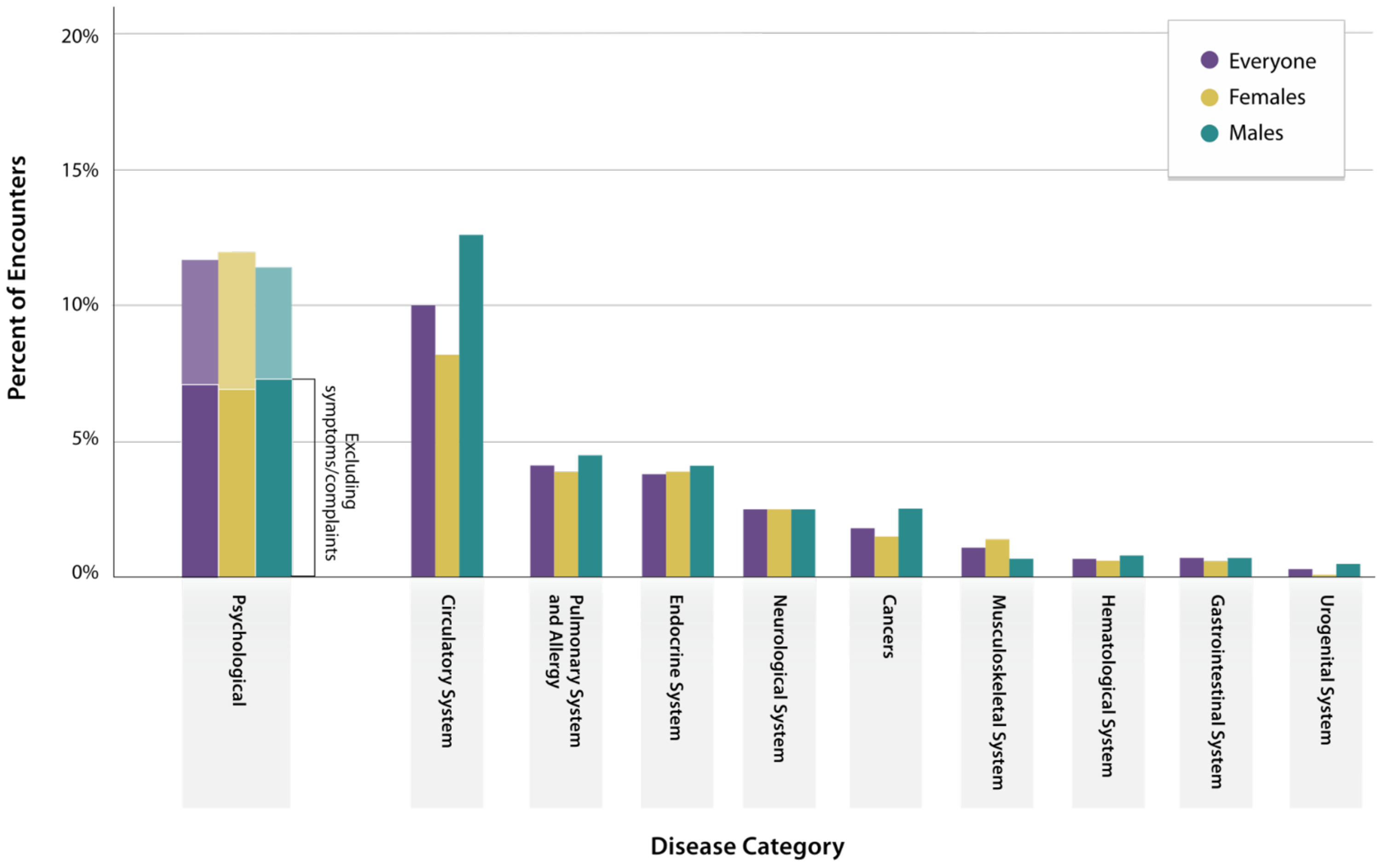

References

Launders N, Kirsh L, Osborn DPJ, Hayes JF. The temporal relationship between severe mental illness diagnosis and chronic physical comorbidity: a UK primary care cohort study of disease burden over 10 years. Lancet Psychiatry 2022;9:725-735.
DOI: 10.1016/S2215-0366(22)00225-5

Momen, NC, Plana-Ripoll, O, Agerbo, E, et al. (2021). Association between Mental Disorders and Subsequent Medical Conditions. N Engl J Med 2020; 382:1721-1731.
DOI: 10.1056/NEJMoa1915784
