# Supplementary material for: A nationwide analysis of 350 million patient encounters reveals a high volume of mental-health conditions in primary care
Source: Nat Ment Health. 2024 Sep 19;2(10):1208–16. doi: 10.1038/s44220-024-00310-5 (PMC11479939; doi:10.1038/s44220-024-00310-5)
Supplement: Supplementary file 2 — Reporting Summary [file 44220_2024_310_MOESM2_ESM.pdf]

nature portfolio

Corresponding author(s): Avshalom Caspi

Last updated by author(s): Mar 29, 2024

Reporting Summary

Nature Portfolio wishes to improve the reproducibility of the work that we publish. This form provides structure for consistency and transparency in reporting. For further information on Nature Portfolio policies, see our Editorial Policies and the Editorial Policy Checklist.

Statistics

For all statistical analyses, confirm that the following items are present in the figure legend, table legend, main text, or Methods section.

n/a	Confirmed	
☐	☒	The exact sample size (*n*) for each experimental group/condition, given as a discrete number and unit of measurement
☐	☒	A statement on whether measurements were taken from distinct samples or whether the same sample was measured repeatedly
☐	☒	The statistical test(s) used AND whether they are one- or two-sided<br>*Only common tests should be described solely by name; describe more complex techniques in the Methods section.*
☒	☐	A description of all covariates tested
☒	☐	A description of any assumptions or corrections, such as tests of normality and adjustment for multiple comparisons
☐	☒	A full description of the statistical parameters including central tendency (e.g. means) or other basic estimates (e.g. regression coefficient) AND variation (e.g. standard deviation) or associated estimates of uncertainty (e.g. confidence intervals)
☒	☐	For null hypothesis testing, the test statistic (e.g. *F*, *t*, *r*) with confidence intervals, effect sizes, degrees of freedom and *P* value noted<br>*Give P values as exact values whenever suitable.*
☒	☐	For Bayesian analysis, information on the choice of priors and Markov chain Monte Carlo settings
☒	☐	For hierarchical and complex designs, identification of the appropriate level for tests and full reporting of outcomes
☒	☐	Estimates of effect sizes (e.g. Cohen's *d*, Pearson's *r*), indicating how they were calculated

Our web collection on statistics for biologists contains articles on many of the points above.

Software and code

Policy information about availability of computer code

Data collection	No software was used
Data analysis	Analyses were performed in SAS v9.4 TS Level 1M4; graphs were created in R using plotly (v4.9.2.2); we have added a link to the code in the manuscript under Code Availability.

For manuscripts utilizing custom algorithms or software that are central to the research but not yet described in published literature, software must be made available to editors and reviewers. We strongly encourage code deposition in a community repository (e.g. GitHub). See the Nature Portfolio guidelines for submitting code & software for further information.

Data

Policy information about availability of data

All manuscripts must include a data availability statement. This statement should provide the following information, where applicable:

- Accession codes, unique identifiers, or web links for publicly available datasets
- A description of any restrictions on data availability
- For clinical datasets or third party data, please ensure that the statement adheres to our policy

The data for this study are primary-care records for entire cohorts of the Norwegian population. Researchers can access the data by application to the Regional Committees for Medical and Health Research Ethics and the data owners (Statistics Norway and the Norwegian Directorate of Health). The authors cannot share these data with other researchers. However, other researchers can contact the authors if they have questions concerning the data.

Research involving human participants, their data, or biological material

Policy information about studies with human participants or human data. See also policy information about sex, gender (identity/presentation), and sexual orientation and race, ethnicity and racism.

Reporting on sex and gender	This population-based study included 4,875,722 individuals: 2,433,978 males; 2,441,744 females. We report on assigned sex as determined at birth in the population registry. We report analysis for the full population and stratified by sex.
Reporting on race, ethnicity, or other socially relevant groupings	This population-based study included 4,875,722 individuals (2,433,978 males; 2,441,744 females) born in Norway between February, 1905 and December, 2017 who were full-time residents in Norway from January 2006 until December 2019 or until they died, as identified in the Norwegian Population Register. We report analyses for the population between 0-100 years, and stratified by age.
Population characteristics	This population-based study included 4,875,722 individuals (2,433,978 males; 2,441,744 females) born in Norway between February, 1905 and December, 2017 who were full-time residents in Norway from January 2006 until December 2019 or until they died, as identified in the Norwegian Population Register.
Recruitment	All residents of Norway are assigned a primary-care physician (PCP). To receive reimbursements, PCPs bill the Norwegian Health Economics Administration, sending diagnosis or reason for the visit.
Ethics oversight	Approval for this study was obtained from the Regional Committee for Research Ethics South East Norway (REK South East) and the Institutional Review Board at Duke University, Campus IRB Protocol 2022-0260. This has been added to the manuscript.

Note that full information on the approval of the study protocol must also be provided in the manuscript.

Field-specific reporting

Please select the one below that is the best fit for your research. If you are not sure, read the appropriate sections before making your selection.

☐ Life sciences ☒ Behavioural & social sciences ☐ Ecological, evolutionary & environmental sciences

For a reference copy of the document with all sections, see nature.com/documents/nr-reporting-summary-flat.pdf

Behavioural & social sciences study design

All studies must disclose on these points even when the disclosure is negative.

Study description	Nationwide population registry study. The data are quantitative.
Research sample	This representative population-based study included 4,875,722 individuals (2,433,978 males; 2,441,744 females) born in Norway between February, 1905 and December, 2017 who were full-time residents in Norway from January 2006 until December 2019 or until they died, as identified in the Norwegian Population Register.
Sampling strategy	The data provide information about ALL individuals born in Norway between 1905-2017 who visited their primary-care physician.
Data collection	Analysis of medical records. All residents of Norway are assigned a primary-care physician (PCP). To receive reimbursements, PCPs bill the Norwegian Health Economics Administration, sending diagnosis or reason for the visit. The researchers were not involved in data collection.
Timing	Data were collected from January 2006 until December 2019.
Data exclusions	Figure 1B shows a flow chart where primary-care records were excluded if they were incomplete, did not involve direct contact with a primary-care physician, or did not involve a medical condition. 160,916,938 of 515,433,229 encounters were deleted.
Non-participation	No participants dropped out. This is a registry study of primary-care encounters in an entire nation.
Randomization	Randomization was irrelevant for this study; we used the entire population.

Reporting for specific materials, systems and methods

We require information from authors about some types of materials, experimental systems and methods used in many studies. Here, indicate whether each material, system or method listed is relevant to your study. If you are not sure if a list item applies to your research, read the appropriate section before selecting a response.

Materials & experimental systems

n/a	Involved in the study
☒	☐ Antibodies
☒	☐ Eukaryotic cell lines
☒	☐ Palaeontology and archaeology
☒	☐ Animals and other organisms
☒	☐ Clinical data
☒	☐ Dual use research of concern
☒	☐ Plants

Methods

n/a	Involved in the study
☒	☐ ChIP-seq
☒	☐ Flow cytometry
☒	☐ MRI-based neuroimaging

Plants

Seed stocks

Report on the source of all seed stocks or other plant material used. If applicable, state the seed stock centre and catalogue number. If plant specimens were collected from the field, describe the collection location, date and sampling procedures.

Novel plant genotypes

Describe the methods by which all novel plant genotypes were produced. This includes those generated by transgenic approaches, gene editing, chemical/radiation-based mutagenesis and hybridization. For transgenic lines, describe the transformation method, the number of independent lines analyzed and the generation upon which experiments were performed. For gene-edited lines, describe the editor used, the endogenous sequence targeted for editing, the targeting guide RNA sequence (if applicable) and how the editor was applied.

Authentication

Describe any authentication procedures for each seed stock used or novel genotype generated. Describe any experiments used to assess the effect of a mutation and, where applicable, how potential secondary effects (e.g. second site T-DNA insertions, mosiacism, off-target gene editing) were examined.
